# Atypical presentation of paroxysmal nocturnal hemoglobinuria treated by eculizumab: A case report: Erratum

**DOI:** 10.1097/MD.0000000000006706

**Published:** 2017-04-21

In the article, “Atypical presentation of paroxysmal nocturnal hemoglobinuria treated by eculizumab: A case report”,^[1]^ which appeared in Volume 96, Issue 12 of *Medicine*, Dr. Régis Peffault de Latour's name appeared incorrectly as Peffault de Latour Régis.
